# Effect of Probiotic Yogurt Supplementation(*Bifidobacterium animalis* ssp. lactis BB-12) on Gut Microbiota of Female Taekwondo Athletes and Its Relationship with Exercise-Related Psychological Fatigue

**DOI:** 10.3390/microorganisms11061403

**Published:** 2023-05-26

**Authors:** Jiang Zhu, Yuping Zhu, Gang Song

**Affiliations:** 1 Southwest University Hospital, Chongqing, 400715, China; zhujiang731@aliyun.com; 2 College of Physical Education, Southwest University, Chongqing 200715, China

**Keywords:** lactis BB-12, athletes, sports mental fatigue, gut microbiota

## Abstract

Objective: The gut microbiota plays a critical role in regulating human health and athletic performance. Probiotic supplementation has been shown to modulate gut microbiota composition and improve exercise performance. This study aimed to investigate the effect of probiotic yogurt supplementation on gut microbiota and its relationship with exercise-related psychological fatigue in female taekwondo athletes. Methods: Twenty female taekwondo athletes were randomly assigned to either a dietary intervention group (DK) or a control group (CK). The athletes’ exercise-related psychological fatigue was measured using the Athlete Burnout Questionnaire (ABQ) before and after an 8-week intervention. High-throughput sequencing was used to profile the gut microbiota, and functional prediction of the microbial community was performed. The effect of the dietary intervention on the athletes’ exercise-related psychological fatigue clearance rate and its relationship with the gut microbiota were explored. Results: (1) The probiotic supplementation of *Bifidobacterium animalis* ssp. lactis BB-12 for 8 weeks significantly increased the ABQ scores of the DK group compared to the CK group (*p* < 0.05). (2) The abundances of *Bifidobacterium*, *Bacteroides*, *Lachnospiraceae*, family _*Lactobacillaceae*, and genus _*Lactobacillus* were significantly higher in the DK group than in the CK group after probiotic supplementation, while *Escherichia coli* was significantly lower in the DK group than in the CK group. (3) The ABQa scores were positively correlated with *Proteus*; ABQb scores were positively correlated with *Streptococcus* and *Enterococcus*; and ABQc scores were positively correlated with *Klebsiella*, *Bacteroides*, and *Streptomyces*. (4) The DK group had significantly higher levels of L-arginine biosynthesis I (via L-ornithine), fatty acid biosynthesis and oxidation, and L-isoleucine biosynthesis III pathways compared to the CK group. Tyrosine degradation I (via 2,3-dihydroxyphenylpropionate) was significantly lower in the DK group than in the CK group. Conclusions: Probiotic yogurt supplementation of *Bifidobacterium animalis* ssp. lactis can promote the clearance of exercise-related psychological fatigue in female taekwondo athletes by upregulating beneficial gut microbiota, inhibiting harmful gut microbiota, and regulating relevant metabolic pathways.

## 1. Introduction

The process from preparation to completion of a competition requires continuous intensive training, which can be tedious and monotonous for athletes. During competitions, athletes experience significant psychological pressure, which can result in post-competition related mental fatigue. Smith [1] proposed that psychological fatigue is a manifestation of mental and emotional distress, characterized by chronic dissatisfaction with previously enjoyed activities due to excessive pressure, resulting in withdrawal. In addition, sports-related psychological fatigue is a critical factor affecting athletic performance. After conducting psychological fatigue intervention experiments on 16 young swimmers, Penna et al. found that the induction of psychological fatigue impaired the athletic performance of young swimmers [2]. Moreover, meta-analysis shows that psychological fatigue impairs the athletic skills of high-level athletes, including technical and decision-making abilities, and is associated with impaired executive function [3]. Furthermore, sports-related psychological fatigue is an important factor affecting endurance performance [4]. Therefore, psychological fatigue has been a research hotspot in the field of sports health.

Eliminating post-competition psychological fatigue can involve various methods, such as interrupting or modifying training, psychological recovery training, and ensuring adequate sleep and nutrition. Among these, scientifically formulated dietary supplements can effectively influence an individual’s emotional and cognitive states. For instance, in probiotic supplementation, research has found that nutritional probiotics can improve an individual’s emotional health, including anxiety, depression, and performance under stress [5,6,7], as well as cognitive function [8,9]. In the population of athletes, probiotic supplementation has also been shown to effectively improve their mood [10], reduce stress [11], enhance cognitive function [12], and consequently improve their athletic performance [13]. Previous research has also demonstrated that adding the subspecies of *Bifidobacterium animalis* ssp. lactis BB-12 to the diet can regulate hypoxia and improve the performance of divers under stressful conditions [14], as well as improve their state anxiety and resilience under pressure (high and low pressure) [15]. Therefore, supplementing with probiotics can promote the clearance of post-competition psychological fatigue by improving emotional, stress, and cognitive function. However, the mechanisms behind this effect are still not clear.

The gut microbiota is a potential mechanism by which probiotics can improve an individual’s cognitive function and emotional health. Probiotics act on the microbiota–gut–brain (MGB) axis by influencing the gut microbiota, which in turn affects an individual’s emotions and cognitive function [16,17,18,19]. The MGB axis is a bidirectional signaling system between the gastrointestinal tract and the central nervous system through spinal cord neurotransmitters and the vagus nerve, which can modulate the brain and behavior by regulating the composition, structure, and diversity of the gut microbiota and transmitting signals to the central nervous system [20]. The gut microbiota regulates neurotransmitters, such as gamma-aminobutyric acid (GABA) [21] and serotonin (5-HT) [22], activates the vagus nerve [23], and affects the hypothalamic–pituitary–adrenal (HPA) axis [24], thus influencing cognitive function. Additionally, studies have found that regulating the gut microbiota and its metabolites can effectively improve mental fatigue [25] and athletic performance [26] in athletes through the MGB axis. The gut microbiota may be an important basis for supplementing probiotics to clear post-competition psychological fatigue in athletes.

Currently, there is limited research on the relationship between gut microbiota and exercise-induced psychological fatigue; particularly, the mechanism of action of probiotic supplementation on post-exercise psychological fatigue in athletes remains unclear. It is hypothesized that probiotic supplements, by modulating the gut microbiota of athletes, could regulate cognitive brain function and emotional state, promote the clearance of exercise-induced psychological fatigue, and act on the MGB axis. In this study, we chose taekwondo athletes who are susceptible to psychological fatigue during high-intensity exercise as the study population. Previous research has shown that *Bifidobacterium animalis* ssp. lactis BB-12 is a probiotic strain that can improve individuals’ emotions (anxiety and depression) and cognitive function [10,19]. Therefore, in this study, *Bifidobacterium* BB-12 was selected as the strain for an 8-week dietary intervention in taekwondo athletes, and the changes in exercise-induced psychological fatigue and their relationship with gut microbiota were observed. This study provides theoretical support for the application of gut microbiota in exercise psychology research and provides a theoretical basis for improving the clearance rate of post-exercise psychological fatigue in athletes.

## 2. Materials and Methods

### 2.1. Participants

After obtaining informed consent, a total of 59 participants were initially enrolled in the study, with 8 participants subsequently withdrawing. The remaining 51 participants were randomly allocated into two groups: 25 participants (DK group) were assigned to the dietary intervention group which received regular training using yogurt with *Bifidobacterium animalis* ssp. lactis BB-12 as a dietary intervention, while 26 participants (CK group) were assigned to the control group which received routine treatment without dietary intervention.

Inclusion criteria were as follows: (1) taekwondo athletes; (2) absence of organic or psychological diseases after physical examination; and (3) no history of intestinal surgery or inflammatory bowel disease. Exclusion criteria were as follows: (1) recent use of antibiotics; (2) occurrence of diarrhea and insomnia in the week before the experiment; (3) long-term consumption of nutrition fiber; and (4) having received training on how to alleviate exercise-related mental fatigue. During the experiment, all subjects received strict dietary and accommodation arrangements. All athletes were informed of the entire experimental process and signed informed consent forms. This study was approved by the Ethics Committee of the School of Physical Education at Southwest University, Chongqing, China.

### 2.2. Experimental Design

**Figure 1:**
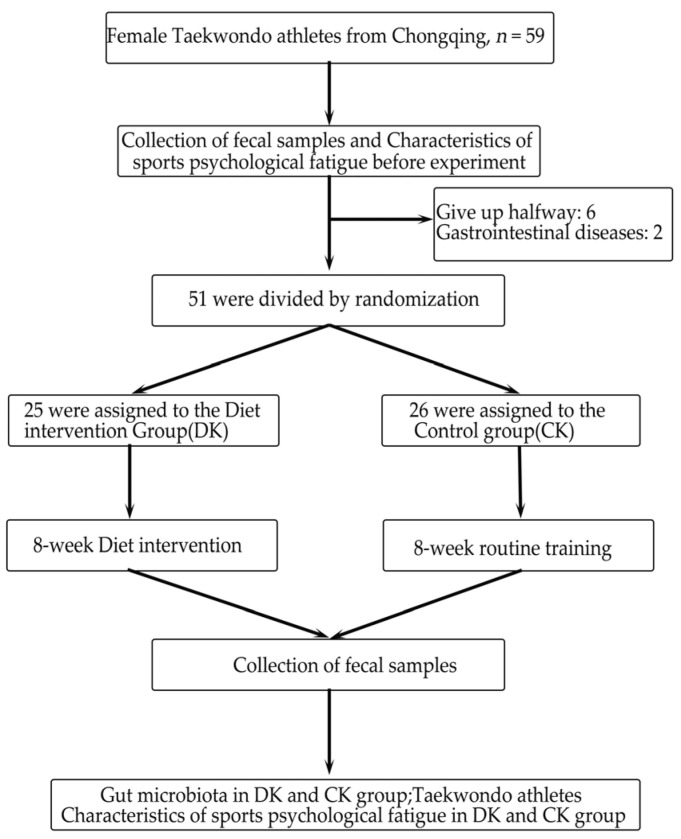
Experiment flowchart.

Firstly, a survey was conducted to collect background information and dietary habits of all taekwondo athletes. Then, after the intervention, the athletes’ gut microbiota and exercise-induced mental fatigue were tested. After an 8-week yogurt intervention, the gut microbiota and exercise-induced mental fatigue characteristics of the two groups of patients were measured (Figure 1).

### 2.3. Sample Collection

Collection of each subject’s feces was placed in a collection box and stored at −80 °C. All stool samples were collected at Southwestern University Hospital.

### 2.4. Yogurt

The yogurt used in this study (produced by Inner Mongolia Yili Industrial Group Co., Ltd., Hohhot, China) contained the subspecies of *Bifidobacterium animalis* ssp. lactis BB-12. The *Bifidobacterium animalis* ssp. lactis BB-12 strain (1 × 10^9^ CFU/100 g) was in accordance with the approval certificate for domestic health food issued by the China National Medical Products Administration (No. 2015B0306). The yogurt was purchased in 250 mL containers, and the remaining yogurt after consumption was measured. The milk cartons for the athletes were numbered, and the remaining yogurt in the cartons was calculated. A cream sampler (Beijing Jinsu Instruments Co., Ltd., Beijing, China) was used to extract the remaining yogurt from the bottle, which was then squeezed into a 25 mL measuring cylinder for measurement. The yogurt intake of each athlete was calculated as 250 mL minus the remaining amount. Every day, one researcher measured the daily yogurt intake of each athlete to calculate the intake of lactis BB-12.

### 2.5. Athletes’ Mental Fatigue

This study used the Athlete Burnout Questionnaire (ABQ) developed by Raedeke and Smith in 2001, which includes 15 items and measures three subscales: emotional/physical exhaustion, reduced sense of accomplishment, and sport devaluation [25]. The questionnaire was distributed to 40 participants, and 40 valid responses were collected. Participants rated each item on a 5-point scale: 1 = never, 2 = seldom, 3 = sometimes, 4 = often, 5 = always. Scores range from 5 to 25, with higher scores indicating greater levels of burnout.

### 2.6. Bioinformatics Analysis of the Gut Microbiota

Stool sample collection and DNA extraction: stool samples were collected using a stool sample collector and stored frozen at −80 °C. DNA extraction was performed using the Fast DNA Stool Mini Kit (Qiagen, Redwood City, CA, USA), and the bacterial DNA concentration was measured using a NanoDrop 2000 spectrophotometer (Thermo Scientific, Waltham, MA, USA).

High-throughput sequencing: Illumina-MiSeq high-throughput sequencing was used to study the bacterial communities in the stool samples. The V3 and V4 regions of the 16S rDNA gene were amplified by PCR and purified, and then the paired-end libraries were constructed and sequenced.

Analysis of species composition: based on the taxonomic results, the species composition of the different groups of athletes at different levels was determined. The Wilcoxon rank-sum test was used to compare the structural differences of the gut microbiota between the two groups of athletes. The LefSe multi-level species difference discriminant analysis method was used to analyze the species differences of the gut microbiota between the two groups of athletes.

### 2.7. Statistical Analysis

Statistical analysis was performed using SPSS version 25.0 to analyze the sports-related psychological fatigue of the diet intervention group and the control group of taekwondo athletes. To analyze the gut microbiota data, Wilcoxon rank-sum test was used to compare the species differences between the two groups of gut microbiota. The LEfSe multi-level species difference discriminant analysis method was used to analyze the species differences in the gut microbiota of the two groups of athletes. Spearman correlation analysis was used to analyze the correlation between gut microbiota and sports-related psychological fatigue. Statistical symbols are expressed as positive and negative standard deviations of the mean ($\bar{x}$ ± SD), with *p* < 0.05 denoting a significant difference and *p* < 0.01 denoting an extremely substantial difference.

## 3. Results

### 3.1. Basic Characteristics of Taekwondo Athletes

All 51 taekwondo athletes were female, and the basic characteristics of the subjects in both groups (including age, years of training exercise level, and intake of food provided by the test) are shown in Table 1.

As seen from Table 1, no significant differences were observed between the two groups of subjects regarding any baseline characteristics for the basic characteristics.

### 3.2. Sports-Related Mental Fatigue Scores in Taekwondo Athletes

During the experiment, athletes who participated were taekwondo participants of the 31st World University Games (delayed due to the COVID-19 pandemic). The athletes’ sports-related psychological fatigue was collected at week 0 and week eight after the athletes’ competition, respectively.

**Figure 2:**
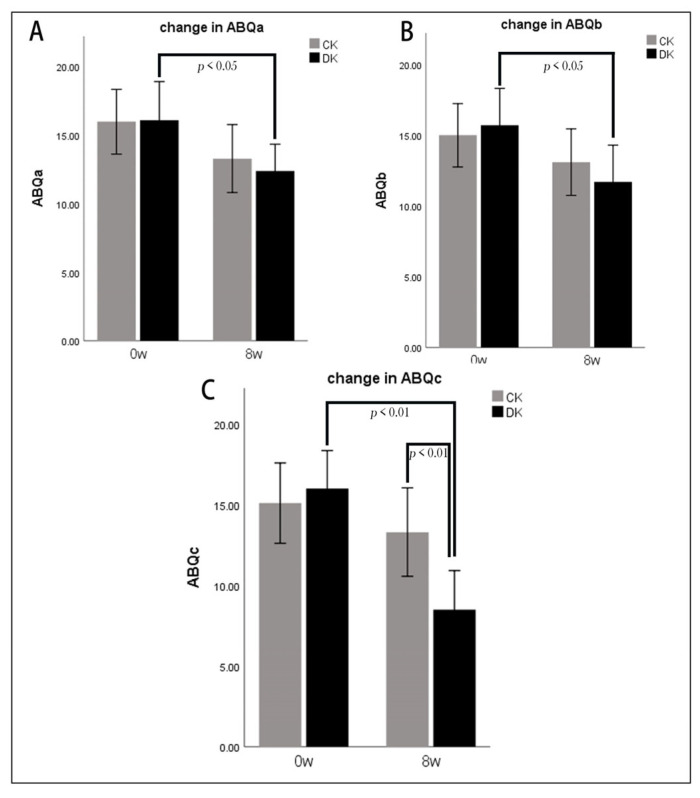
Athletes’ psychological evaluation scores of sportiness before and after dietary intervention. Panel (**A**) shows the change before and after the dietary intervention for ABQa (reduced sense of achievement); panel (**B**) shows the change before and after the dietary intervention for ABQb (emotional and physical exhaustion); panel (**C**) shows the change before and after the dietary intervention for ABQc (negative evaluation of locomotion); data between groups were analyzed using the Wilcoxon paired signed-rank test; *p*-values are shown in each panel. *p* < 0.05 = significantly different. Data are expressed as mean ± standard deviation ($\bar{x}$ ± SD).

As shown in Figure 2, compared to week 0, the athletes’ ABQascores significantly decreased (*p* < 0.05) after 8 weeks of dietary intervention, including emotional and physical exhaustion (ABQb) and negative evaluation of sports (ABQa). After 8 weeks, the ABQc score of the DK group was significantly lower than that of the CK group (*p* < 0.01).

### 3.3. Differences in Intestinal Microbiota between the DK and CK Groups after Dietary Intervention

**Figure 3:**
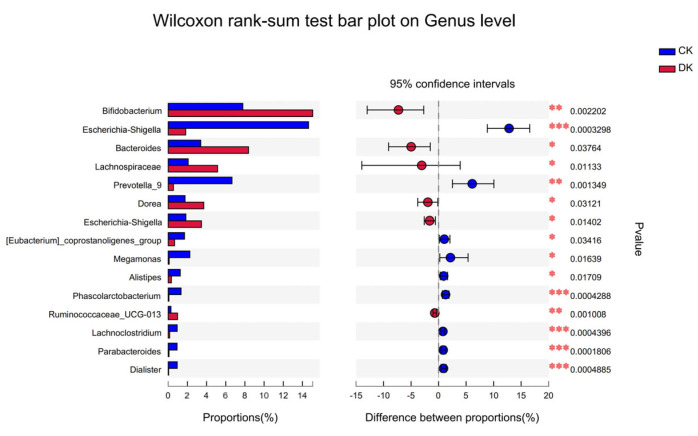
Differences between species groups in gut microbiota. The vertical axis (left) shows the names of bacteria at the genus level. Each column on the vertical axis represents the mean relative abundance of the genus in each sample group. Red represents the dietary intervention group, and blue represents the control group. The left horizontal axis represents the proportion of each type of bacteria. The right horizontal axis indicates the difference between groups. The dot’s color indicates the group with greater horizontal abundance, and the I-shaped interval indicates the difference’s upper and lower limits. * *p* < 0.05 is a significant difference, and ** *p* < 0.01 is highly significant, and *** *p* < 0.001 is highest significant.

As shown in Figure 3, significant differences were observed between the two groups of subjects at the genus level after 8 weeks of dietary intervention. Specifically, *Bifidobacterium*, *Prevotella*, and *Lachnospiraceae* were significantly increased in the DK group, while *Escherichia–Shigella* was significantly decreased in the control group.

**Figure 4:**
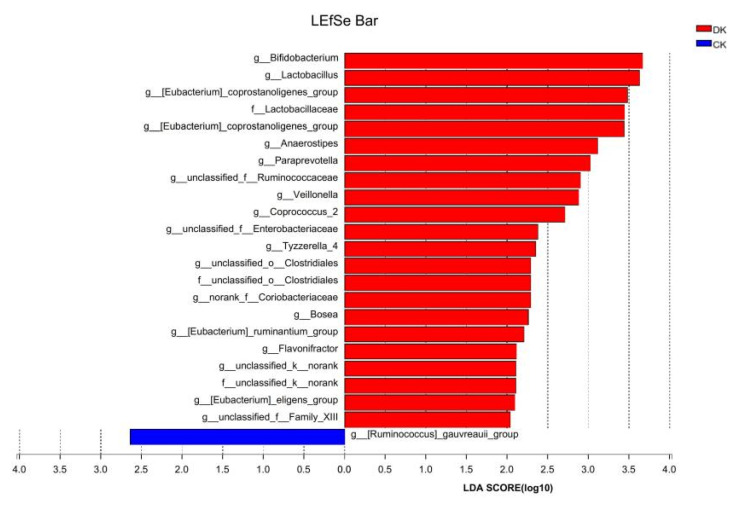
Analysis of LEfSe in the DK group and CK group after dietary intervention. LDA discriminant bar graphs count the microbial taxa with significant effects in multiple groups and the LDA scores obtained by LDA analysis (linear regression analysis), with larger LDA scores representing a greater effect of species abundance on differential effects. The vertical axis shows the names of bacteria at the genus and family levels. Each column on the vertical axis represents the mean relative abundance of the genus in each sample group. Red represents the dietary intervention group, and blue represents the control group. The horizontal axis represents the proportion of each type of bacteria.

Differences in the abundance of gut microbiota at different taxonomic levels between the two groups are shown in Figure 4. After the dietary intervention, the abundance of family *Bifidobacteriaceae*, genus *Bifidobacterium* (g__*Bifidobacterium*) in the DK group and family *Lactobacillaceae*, genus *Bifidobacterium* (f__*Lactobacillaceae*_g__*Bifidobacterium*) and genus *Lactobacillus* (g__*Lactobacillus*) in the DK group significantly increased (*p* < 0.05).

### 3.4. Relationship between Exercise Psychological Fatigue and Gut Microbiota after Dietary Intervention

**Figure 5:**
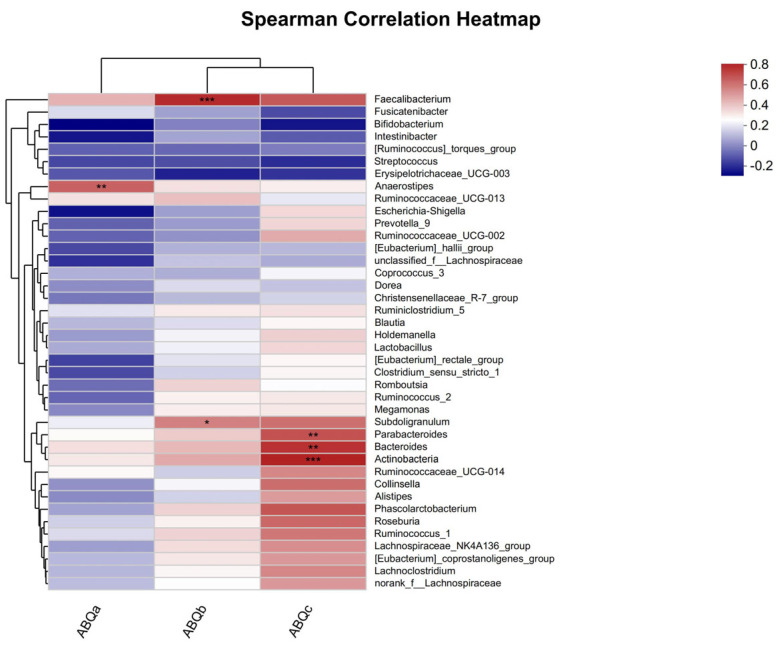
Relationship between ABQ and gut microbiota. The *x*-axis and *y*-axis are environmental factors and gut microbiota species, respectively, and the associated R- and *p*-values can be calculated. The figure shows R-values in different colors, with a positive correlation in red. Blue is a significant negative correlation. * = *p* < 0.05, ** = *p* < 0.01, *** = *p* < 0.001. The legend on the right side shows the color intervals of the different R values. abqa = reduced achievement, abqb = emotional and physical exhaustion, abqc = negative evaluation of exercise.

As shown in Figure 5, there were significant correlations between ABQ scores and the relative abundance of gut microbiota in both groups after 8 weeks of dietary intervention. Specifically, ABQa was significantly positively correlated with *Anaerostipes*; ABQb was significantly positively correlated with *Streptococcus* and *Bacteroidales* S24-7; and ABQc was significantly positively correlated with *Hydrogenophaga*, *Parabacteroides*, and *Actinobacteria*.

### 3.5. Metabolic Pathways of the Gut Microbiota in the Dietary Intervention and Control Groups

**Figure 6:**
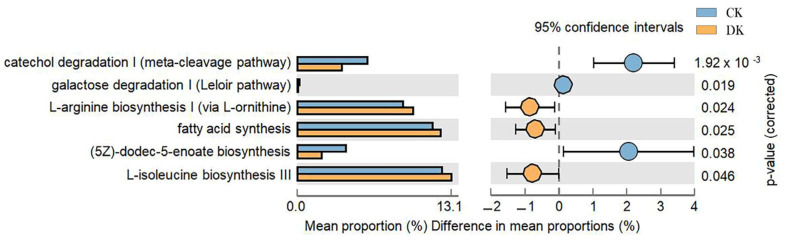
Metabolic pathway differences in gut microbiota using PICRUSt2.0 to predict the MetaCyc pathway between the gut microbiota of the DK group and the CK group of taekwondo athletes after dietary intervention. Each column on the vertical axis represents the mean level of this pathway in each sample group. Yellow represents the exercise group, and blue represents the control group. The horizontal axis on the left represents the proportion of each metabolic pathway. The horizontal axis on the right indicates the difference between the different groups. The middle area is the difference in the percentage of family abundance between the two groups within the set confidence interval. The color of the dots indicates the group with greater horizontal plenty, and the I-shaped intervals of the dots indicate the upper and lower limits of the differences.

As shown in Figure 6, the analysis of differences in gut microbiota metabolic pathways reveals six differences between the DK and CK groups after 8 weeks of dietary intervention. Specifically, the L-arginine biosynthesis I (via L-glutamate), fatty acid biosynthesis and oxidation, and L-isoleucine biosynthesis III pathways were significantly higher in the DK group compared to the CK group. Meanwhile, the catecholamine degradation I (ortho pathway) was significantly lower in the DK group compared to the CK group.

## 4. Discussion

### 4.1. Effect of Bifidobacterium animalis ssp. lactis BB-12Supplementation on Motor Mental Fatigue Clearance in Athletes

Psychological fatigue in athletes (including decreased attention, motivation, and willpower) is an important factor that affects athletic performance [26]. Additionally, an imbalance between chronic fatigue and insufficient recovery can lead to adverse consequences, such as overtraining syndrome and inadequate recovery in athletes, ultimately resulting in long-term decreases in factors such as athletic ability and well-being, thereby impacting athletic performance [27]. Long-term high-intensity training is a potential cause of psychological fatigue in athletes; for example, Hu et al. [28] found that professional weightlifting training led to deep fatigue in the central nervous system, skeletal muscles, and respiratory system of athletes, with a long recovery time. Additionally, through research, Gould et al. [29,30] found that both physical and psychological stress were important factors leading to psychological fatigue in tennis players. It has been found that supplementing with probiotics can significantly improve individuals’ mood [10], cognition [19], and recovery from fatigue [31]. *Bifidobacterium animalis* ssp. lactis BB-12 is a subspecies of probiotic-rich yogurt belonging to the same probiotic group. However, there are few studies on the effects of *Bifidobacterium animalis* ssp. lactis BB-12 supplements on the mental health, cognitive function, and fatigue clearance rate of this specific athlete population. Based on this, this study explores whether dietary intervention can improve post-match psychological fatigue in taekwondo athletes through intervention with *Bifidobacterium animalis* ssp. lactis BB-12 yogurt.

The present study utilized scales to measure the post-exercise psychological fatigue recovery of taekwondo athletes before and after an 8-week dietary intervention. The results showed that compared to the CK group, the ABQa, ABQb, and ABQc scores of taekwondo athletes were significantly reduced. It has been found that continuous supplementation with plant-derived *Lactobacillus plantarum* PS128 for 30 days can effectively reduce individual fatigue, brain wave activity, and cortical excitability, as well as improve depression [32]. In addition, supplementation with a mixture of probiotics (containing fermented *Lactobacillusfermentum* LF16, *Bifidobacterium lactis* LR06, *Lactobacillus plantarum* LP01, and *Bifidobacterium animalis* subsp. lactis BL04) significantly improved the mood state (e.g., depression and anger) and sleep quality of the subjects, as well as improving their mental health [33]. *Bifidobacterium longum* BB536 can reduce the neuroreactivity during social stress by regulating and increasing the restful neural activity associated with energy and decreasing mental fatigue [34]. In addition, our previous study found that *Bifidobacterium animalis* ssp. lactis can improve the state anxiety and exercise performance of divers at different pressure levels [15]. Probiotic supplementation can improve the post-exercise psychological fatigue of taekwondo athletes, which may be due to the ability of *Bifidobacterium animalis* ssp. lactis to improve emotions, cognition, regulate brain activity, and further promote the clearance of post-exercise psychological fatigue in taekwondo athletes.

### 4.2. Effect of Bifidobacterium animalis ssp. Lactis BB-12 Supplementation on the Intestinal Microflora of Athletes

Probiotics are microorganisms isolated from the intestinal microbiota that exert health-promoting effects on the host mainly through mechanisms such as interference with potential pathogens, improvement of barrier function, immune regulation, and upregulation of neurotransmitter production, which are closely related to the MGB [35]. Furthermore, studies have shown that probiotic supplementation can effectively improve tissue health, athletic performance, and exercise capacity in athletes [26]. Dietary supplementation with the plant-derived *Lactobacillus plantarum* TWK10 increased grip strength and swimming time in mice, as well as the percentage of type I muscle fibers in the gastrocnemius muscle [36]. Additionally, probiotic supplementation can improve recovery after exercise, as evidenced by a significant decrease in creatine kinase (CK) levels in IRONMANtriathletes supplemented with *Lactobacillus plantarum* PS128 three hours after exercise [37]. CK levels are an important biochemical marker of post-exercise fatigue. Probiotic supplementation also enhances the body’s antioxidant capacity [37], reduces myeloperoxidase activity, and increases sulfhydryl oxidase activity [38], suggesting that probiotic supplementation can alleviate exercise-induced oxidative stress and related inflammation. Probiotic supplementation is helpful for the health of the host, particularly for improving the athletic performance of athletes and promoting their recovery from exercise. However, it has been reported that supplementation with *Bifidobacterium animalis* ssp. lactis BB-12 may further improve the cognitive and emotional states of athletes by regulating the gut microbiota, thus facilitating the clearance of post-competition psychological fatigue [15]. Based on this, the present study investigated the effects of an 8-week dietary intervention on the clearance rate of post-competition psychological fatigue in athletes and its relationship with the gut microbiota, aiming to fill a gap in the literature.

In this study, we analyzed the differences in the gut microbiota between the DK and CK groups at the genus level and found that supplementing with *Bifidobacterium animalis* ssp. lactis BB-12 for eight weeks significantly improved the levels of *Bifidobacterium*, *Bacteroides*, and *Lactobacillaceae* in taekwondo athletes. There are gender differences in levels of depression, cognitive stress, and anxiety. Women tend to exhibit higher levels compared to men [39,40,41,42]. *Bifidobacterium* and *Clostridium*, which are closely associated with these psychological and cognitive disorders, may be potential therapeutic targets, especially in the female population [43]. In addition, the genus *Clostridium* is associated with various metabolic pathways, including arginine metabolism, proline metabolism, and fatty acid biosynthesis, and is linked to a broad network of functional connections that mediate the relationship between *Clostridium*-associated metabolic pathways and cognition. The abundance of *Lactomycopin* in patients with Alzheimer’s disease (AD) is significantly reduced, and the likelihood of beta-amyloid protein and phosphorylated tau (*p*-tau) positivity is higher, which may be due to the reduction in *Lactomycopin* abundance leading to a decrease in short-chain fatty acid (SCFA) generation [44]. SCFAs are important regulatory factors in the gut-brain axis, and play an important role in the MGB axis [45,46,47].

Furthermore, *Escherichia coli–Shigella*, which was significantly reduced in the DK group, has been shown to be closely associated with mental fatigue. It has been found to significantly increase in cognitive disorders, such as post-stroke cognitive impairment [48], Parkinson’s disease [49], and post-traumatic stress disorder [50], and may be an important pathogenic factor related to the onset of psychiatric and psychological disorders. The increased abundance of the genus *Clostridium* and *Lactobacillaceae*, as well as the decreased abundance of *Escherichia coli–Shigella* in this study, may improve the cognitive function of taekwondo athletes by mediating the MGB axis, thereby promoting the recovery of exercise-induced mental fatigue.

Further analysis using Linear Discriminant Analysis Effect Size (LEfSe) was employed to investigate the differences in gut microbiota abundance between two groups with different levels. The results revealed that the abundance of g_*Bifidobacterium*, f_*Lactobacillaceae*, and g_*Lactobacillus* in the DK group was significantly higher than that in the CK group. As previously mentioned, g_*Bifidobacterium* is closely related to depression, neurocognition, stress, and anxiety, and may serve as a potential target for the treatment of psychological and cognitive disorders [43]. g_*Lactobacillaceae* (at the family or genus level) is also closely related to emotions and cognition. It has been found that the addition of probiotics, such as *Lactobacillus acidophilus* W37, *Lactobacillus brevis* W63, *Lactobacillus paracasei*, *Lactobacillus fermentum* W56, *Lactobacillus salivarius* W24, and others, significantly reduces overall cognitive responses to sad emotions, mainly due to a decrease in aggressive thinking [51]. In addition, dietary supplementation of *Lactobacillus* in a group of athletes improved their anxiety, stress, and emotional levels, and increased aerobic metabolism [10].

### 4.3. The Relationship between Gut Microbiotaand Exercise Mental Fatigue

The gut microbiota plays a significant role in regulating host cognitive function [52] and emotions (including depression [53] and anxiety [54], among others), influencing host mental health through the complex MGB axis system. Probiotics have been shown to effectively modulate the composition and diversity of the gut microbiota and regulate mental health (including stress and mental disorders [55], depression, and anxiety [5], among others). Based on this, this study analyzed the correlation between the gut microbiota of taekwondo athletes and exercise-related mental fatigue. It was found that there were significant differences in the composition of the gut microbiota between the ABQ and the taekwondo athletes who supplemented their diet with *Bifidobacterium animalis* ssp. lactis BB-12for 8 weeks. ABQa was significantly positively correlated with *Anaerostipes*; ABQb was significantly positively correlated with *Streptococcus* and *Ruminococcus*; and ABQc was significantly positively correlated with *Parabacteroides*, *Bacteroides*, and *Streptomyces*.

Li et al. [56] conducted a non-public trial over 105 days and found that anesthesia was significantly associated with negative emotions such as anxiety, depression, and anger. Meta-analysis showed that anemia drugs significantly increased the risk of depression in patients with depression [57]. This is similar to the results of this study, where a decline in performance (ABQa) is a negative emotion that is significantly positively correlated with *Anaerostipes*, indicating that the athletes’ performance declined after dietary intervention. In addition, the abundance of *Akkermansia* and *Coprococcus* in the gut microbiota of highly fatigued patients was significantly lower than that in normal fatigued patients. This may be because these two genera are important bacteria that produce SCFAs, confirming our research. This study found that supplementing the diet with *Bifidobacterium animalis* ssp. lactis BB-12 significantly reduced athletes’ emotions and physical fatigue (ABQb), and was positively correlated with *Ruminococcus* and *Streptococcus*. In the Crohn’s disease mouse model, ester-induced depression-like behavior was significantly reduced by *Parabacteroides* [58]; the abundance of *Parabacteroides* and *Streptomyces* was significantly higher in AD patients [59]. In this study, it was found that negative evaluation of exercise (ABQc) was significantly positively correlated with *Parabacteroides*, *Bacteroides*, and *Streptomyces*, indicating that dietary intervention reduced the abundance of these bacterial groups and reduced athletes’ negative evaluation of exercise.

Further use of PICRUSt2.0 was applied to predict the differences in gut microbiota metabolic pathways between DK and CK athletes. It was found that after 8 weeks of supplementing with *Bifidobacterium animalis* ssp. lactis BB-12, DK athletes showed significantly higher levels of L-arginine biosynthesis I (via L-ornithine), fatty acid synthesis and oxidation, and L-isoleucine biosynthesis III pathways than CK athletes. In addition, the catechol degradation I (meta-cleavage pathway) in DK group was significantly lower than in the control group. Studies have shown that dietary supplementation with arginine improves individual accuracy, decision-making ability, and reaction time during agame [60]. Arginine also has a significant therapeutic effect on aging, cognitive decline, and depression induced by psychological and social stress [61]. This confirms the findings of this study that supplementing with *Bifidobacterium animalis* ssp. lactis BB-12 improves athlete performance decline (ABQa) by up-regulating the biosynthesis pathway of arginine. Furthermore, fatty acid synthesis and oxidation are closely related to cancer-related fatigue, and the down-regulation of the fatty acid synthesis and oxidation pathway is an important cause of high levels of fatigue in cancer patients [62]. This study found that supplementing with *Bifidobacterium animalis* ssp. lactis BB-12 can improve the emotional and physical fatigue (ABQb) and fatigue scores of taekwondo athletes. It was also found that L-*isoleucinebiosynthesis* is associated with moderate depression, and the biosynthesis pathway of L-*isoleucine* is significantly reduced in women with mild depression compared to those with moderate depression [63]. Consistent with the results of this study, dietary intervention improved the negative evaluation of exercise (ABQc) in athletes by enhancing L-*isoleucine* biosynthesis pathways. In addition, this study found that after dietary supplementation, catechol degradation I was lower than in the control group. Catechol is an endocrine brain secretion related to desire and sensation, and it transmits excitement and pleasure [64]. In this study, the catechol degradation in the DK group was lower than in the CK group, indicating that the DK group was protected from catechol degradation after dietary intervention. This improved the athletic mental fatigue of the athletes.

In conclusion, dietary supplementation with *Bifidobacterium animalis* ssp. lactis effectively improved the richness and diversity of gut microbiota and metabolic pathways, thereby improving the recovery of taekwondo athletes from post-competition exercise-related mental fatigue, which is closely related to cognitive function, emotion, and mental health.

## 5. Conclusions

The supplementation of *Bifidobacterium animalis* ssp. lactis BB-12 containing yogurt wasobservedto improve exercise-induced fatigue recovery in female taekwondo athletes. This effect may be attributed to the modulation of beneficial and harmful gut microbiota, which in turn affects relevant metabolic pathways.

## Figures and Tables

**Table 1:** Basic characteristics of taekwondo athletes.

$\mathrm{Content}\;(\overset{–}{x}$ ± S)	CK	DK	*P* Sig. (Two-Tail)
Age (year)	22.10 ± 0.88	22.50 ± 0.83	0.412
Training years (year)	4.82 ± 0.52	5.02 ± 0.64	0.660
Sports level (grade)	2.05 ± 0.36	2.14 ± 0.28	0.538
Food intake rate (%)	98.2 ± 6.12	97.8 ± 4.29	0.410

DK = diet intervention group; CK = control group. *P* is the value of independent samples and the two-tailed *t*-test for between-group analysis of the CK and DK groups.

## Data Availability

The data are not publicly available due to privacy for athletes.
